# EasyNAT Malaria: a simple, rapid method to detect *Plasmodium* species using cross-priming amplification technology

**DOI:** 10.1128/spectrum.00583-24

**Published:** 2024-06-13

**Authors:** Liu Dong, Qianqian Xu, Linjie Shen, Ruoshui Cao, Xuan Deng, Jian Chen, Haoqin Jiang, Ming Guan

**Affiliations:** 1 Department of Laboratory Medicine, Huashan Hospital, Fudan University, Shanghai, China; Montefiore Medical Center and Albert Einstein College of Medicine, Bronx, New York, USA

**Keywords:** malaria, diagnosis, EasyNAT Malaria Assay, rapid detection

## Abstract

Malaria infection remains a serious threat to human health worldwide. Rapid and accurate detection technology is crucial for preventing malaria transmission and minimizing damage. We aimed to establish and validate a new rapid molecular detection method for malaria, called EasyNAT Malaria Assay, targeting *Plasmodium vivax*, *Plasmodium falciparum*, *Plasmodium ovale*, and *Plasmodium malariae*. The analytical performance of EasyNAT Malaria Assay was determined using positive materials. We identified 42 clinical samples as malaria positive and 95 negative samples. Each sample was examined by four methods: light microscopy, rapid diagnostic test, EasyNAT Malaria Assay, and digital PCR. Diagnostic accuracy and clinical performance were evaluated. The limit of detection (LOD)_95%_ of EasyNAT Malaria was consistently 40 parasites/mL. It specifically amplified *Plasmodium* and performed with reliable repeatability and reproducibility. In 137 clinical samples, EasyNAT Malaria detected four more positive samples than microscopic examination and two more positive samples than rapid diagnostic test (RDT). One clinical sample was positive only under digital PCR. However, no significant differences statistically in sensitivity or specificity were observed. Compared with microscopy, the total, positive, and negative concordance rates of EasyNAT were 97.08%, 100%, and 95.79%, respectively. Enhanced diagnostic accuracy of EasyNAT Malaria in patients who had taken anti-malarial medication before their clinical appointment was observed. The EasyNAT Malaria Assay has good detection efficiency for clinical samples, presents a promising molecular detection tool in clinical practice, and is particularly suitable for rapid screening of high-risk populations in the emergency room.

**IMPORTANCE:**

This study established and validated EasyNAT Malaria Assay as a promising molecular detection tool for malaria screening of high-risk populations in clinical practice. This novel isothermal amplification method may effectively facilitate the rapid diagnosis of malaria and prevent its transmission.

## INTRODUCTION

China was certified malaria-free in 2021 with no indigenous malaria for five consecutive years. However, according to the WHO’s *World Malaria Report 2022,* approximately 247 million malaria cases were reported in 84 malaria-endemic countries globally in 2021, an estimated 59.2 of 1000 people worldwide are at risk of getting malaria infection, and the global malaria mortality rate (deaths per 100,000 population at risk) increased to 14.8 in 2021.

Currently, the focus has shifted to the prevention and control of imported malaria cases. A total of 436 malaria cases were reported between 2016 and 2019 in Shanghai, and 95.18% of them were imported (1). Complete laboratory testing methods are the first line of defense to prevent malaria re-establishment. The common laboratory detection methods for malaria include microscopic examination, rapid diagnostic test (RDT) targeting *Plasmodium* antigen, and polymerase chain reaction (PCR)-based nucleic acid testing (2, 3). Microscopic reading of thick and thin blood smears is recognized as the gold standard for malaria diagnosis. Although it is cost-saving, it is an extremely experience-dependent and time-consuming method. Currently, RDT is the most widely used rapid detection methodology. It has been reported that approximately 3.5 billion RDTs were sold globally between 2010 and 2020, mostly in sub-Saharan African countries. However, due to the limited sensitivity and specificity of immunochromatography and the presence of Pfhrp2/3 gene-deleted *Plasmodium falciparum* parasites, the reliability of RDT diagnosis is dubious in low-percent parasitemia infections of non-falciparum species and Pfhrp2/3 gene-deleted *P. falciparum* parasites. Given that malaria causes significant morbidity and mortality worldwide, there is a compelling and strong clinical demand for a rapid, convenient, and precise molecular method for laboratory diagnosis of malaria, especially when there are no trained parasitologists available.

In this study, we focused on a rapid detection technique called EasyNAT Malaria Assay (Ustar Biotechnologies Ltd, Hangzhou, China) for the molecular detection of *Plasmodium* species, including *P. vivax*, *P. falciparum*, *P. ovale*, and *P. malariae*. The EasyNAT Malaria Assay was carried out based on cross-priming amplification technology (CPA), a novel isothermal amplification technique, targeting 18S ribosomal RNA. The amplification of the target sequence at a constant temperature (58–63°C) through multiple cross-linked primers and probes. 5(6)-carboxyfluorescein (FAM) dye-labeled probes were used to detect the amplification products in CPA.

The EasyNAT Malaria Assay can be carried out by using preloaded reagents in a single cartridge that accommodates DNA extraction, DNA purification, and target gene amplification in an environment with an electrical supply. Only 50 μl fingerstick blood is adequate to complete the sample testing, and the whole detecting process is easily performed. No trained parasitologists or experienced inspectors are necessarily required. After upgrading the original equipment for COVID-19, all the UC0108 nucleic acid analyzers (Ustar Biotechnologies Ltd) in our laboratory can be used for new malaria parasite detection through a specific reaction cartridge. We aimed to assess the accuracy of this novel isothermal amplification method in clinical settings.

## MATERIALS AND METHODS

### Patient enrollment and sample collection

Whole blood samples used in this study were obtained from patients admitted to Huashan Hospital through venous blood collection in vacuum (EDTA anticoagulant) tubes. We included 42 clinical samples identified as malaria positive by microscopic examination and 95 negative samples. All whole blood samples were divided in separate sterile tubes and stored in a −20°C freezer, avoiding repeated freeze–thaw cycles.

### Blood smear, RDT, and droplet digital PCR

Thick blood smears and thin smears under microscopy were adopted as the gold standard for malaria diagnosis. RDTs were carried out by BinaxNOW Malaria rapid diagnostic tests (Abbott, Maine, USA). Digital PCR (dPCR) was performed on a DropDx-2044 digital PCR system (RainSure Scientific, Suzhou, China). The three methods mentioned above were conducted according to the manufacturer’s instructions, and the detailed procedures were described in our previous article (4). The entire detection processes take approximately 20 min for RDT and 4 hours for dPCR.

### Malaria cartridge, primers, and probe used in EasyNAT Malaria Assay

The EasyNAT Malaria Assay (Ustar Biotechnologies Ltd) was conducted on a UC0108 nucleic acid analyzer (Ustar Biotechnologies Ltd). Subsequently, 50-µL whole blood and 2-mL lysis solution (containing magnetic beads) were added into a specific reaction cartridge, and the entire procedure of nucleic acid lysis, extraction, elution, and amplification was completed in one tube. The specific primers and probes (listed in Table 1) were pre-added in the left and right chambers of the cartridge. The functional area distribution and cross-sectional view were described by Chen et al. (5).

**TABLE 1:** The primers and probe were designed to target the 18S ribosomal RNA

Item	Sequence(5’~3’)	Role
Mal-18S-2-FB	ACTAAAAACGGCCATGCA	Forward primer
Mal-18S-2-AP1	TCACCATCCAAGAAATCAAGA	Accelerated primer 1
Mal-18S-2-AP2	TCAATCCTACTCTTGTCTTAAACT	Accelerated primer 2
Mal-18S-2-OP	CAAATTAAGCCGCAAGCTCCAC	Outer primer
Mal-18S-2-IP1	CCTGGTGGTGCCCTTC	Internal primer
Mal-18S-2-CPR	CAAATTAAGCCGCAAGCTCCACCGAGAAAGTTAAAAGAATTGACG	Cross primer
Mal-18S-2-RB	CAAAGTCTTTGGGTTCTGG	Reverse primer
Mal-18S-2-MB	5′-FAM-cgcgcTGCCTGGTGGTGCCCTTCgcgcg-BHQ1-3’	Probe labeled

### EasyNAT Malaria Assay

The detection process started with scanning the unique two-dimensional code on the cover of each detection cartridge and entering the sample ID into the analyzer. The cartridge containing the whole blood sample and nuclear acid extraction solution was mixed well and inserted into the chamber. After starting the instrument, the instrument automatically conducted the subsequent steps in the next 50 min: DNA extraction, DNA purification, target gene amplification, and detection. The results (Tt value and amplification curve), as displayed on the screen, were presented as four Tt values (two for the target gene and two for internal reference genes in the left and right cartridge chambers) with the final result (positive, negative, or invalid).

### Analytical sensitivity and specificity of EasyNAT Malaria Assay

The limit of detection (LOD) was assessed using standardized material for *P. vivax* (National Institutes for Food and Drug Control, Lot 230038–20140502) and *P. falciparum* (National Institutes for Food and Drug Control, Lot 230007–20140501). As for *P. ovale* and *P. malariae*, because no commercial standardized products were available, clinical samples artificially quantified by digital PCR were used. A series of whole blood samples of each malaria species with different gradients was repeatedly analyzed 20 times. The detailed sample concentrations were as follows: 1000, 500, 200, 100, 40, and 20 parasites/mL. The lowest concentration at which 95% of samples were detectable was regarded as the LOD of various species (LOD_95%_).

To investigate analytical sensitivity, positive samples of each *Plasmodium* species and negative samples were re-analyzed five times in five consecutive days. Here, low-concentration samples (approximately 40–80 parasites/mL) were selected for repeat testing, which is considered more suitable for most clinical realities.

Three types of blood parasitic infections other than malaria (*Babesia microti*, *Trypanosoma*, and *Clonorchis sinensis*), five viral infections (Dengue virus, Influenza A virus, Influenza B virus, Respiratory syncytial virus, and SARS-CoV-2 coronavirus), and six other pathogenic infections (*Klebsiella pneumoniae*, *Streptococcus pneumoniae*, *Escherichia coli*, *Staphylococcus aureus*, *Mycoplasma pneumoniae*, and *Chlamydia pneumoniae*) were used to evaluate analytical specificity. Each sample was tested thrice. The results that were negative in all three replicates were considered accurate.

### Diagnostic sensitivity and specificity of EasyNAT Malaria Assay

Forty-two clinical samples identified as malaria positive by microscopic examination (including 35 *P. falciparum*, two *P. vivax*, three *P. ovale*, and two *P. malariae* samples) collected from 2019 to 2023 were re-analyzed to test the diagnostic sensitivity performance of the EasyNAT Malaria Assay. Another 12 whole blood samples from healthy individuals, 20 samples from patients with fever for reasons other than malarial infection, 8 samples from patients with *B. microti, Trypanosoma*, and *C. sinensis* infections, and 55 samples from those patients who were clinically suspected to have malaria but were eventually negative under microscopic examination were also included.

### Statistical analyses

SPSS Statistics (IBM, Armonk, USA) were used for statistical analysis and graph construction. The differences between the detection rates of the different methods were tested using χ^2^ test. A *P* < 0.01 was considered statistically significant. The total, positive, and negative concordance rates were calculated as follows: total concordance rate  =  100%  ×  [(both positive+both negative) / total samples], positive concordance rate  =  100%  ×  [both positive / (both positive+comparision method positive but EasyNAT negative)], and negative concordance rate  =  100%  ×  [both negative / (both negative+comparision method negative but EasyNAT positive)].

## RESULTS

### Analytical sensitivity and specificity

The detection limit of the EasyNAT Malaria Assay was assessed using serial dilution of whole blood samples, and the LOD_95%_ was consistently 40 parasites/mL for four *Plasmodium* species (Table 2).

**TABLE 2:** Detection limit of the EasyNAT Malaria Assay

Concentration of *P. falciparum*-positive samples (parasites/mL）	Positive rate	Concentration of *P. vivax*-positive samples (parasites/mL）	Positive rate
1000	20/20	1000	20/20
500	20/20	500	20/20
200	20/20	200	20/20
100	20/20	100	20/20
40	19/20	40	20/20
20	10/20	20	10/20

Concentration of *P. malariae*-positive samples (parasites/mL）	Positive rate	Concentration of *P. ovale*-positive samples (parasites/mL）	Positive rate
1000	20/20	1000	20/20
500	20/20	500	20/20
200	20/20	200	20/20
100	20/20	100	20/20
40	20/20	40	19/20
20	14/20	20	12/20

To investigate the precision parameters of the EasyNAT Malaria Assay, samples with a low concentration of each *Plasmodium* species (approximately 40–80 parasites/mL) and negative samples were analyzed five times in five consecutive days. All 25 repeated detections for each *Plasmodium* species presented regular amplification curves and were tested positive, and all negative samples remained negative, indicating reliable repeatability and reproducibility.

Furthermore, the EasyNAT Malaria Assay specifically amplified *Plasmodium* samples but did not amplify nucleotide sequences from other types of pathogens. Analysis of the 14 pathogens detected showed no positive results, indicating a high specificity.

### Clinical performance

A total of 137 clinical samples were analyzed using the EasyNAT Malaria Assay, blood smear, RDT, and dPCR. Three samples were positive only by EasyNAT and dPCR, one sample was positive only by dPCR, and two samples were negative only under light microscopy (Table 3). The EasyNAT Malaria Assay detected more positive cases (33.58%, 46/137) than microscopic examination (30.66%, 42/137) and RDT (32.12%, 44/137), but the difference was not significant (*P* = 0.605, χ^2^ = 0.268; *P* = 0.797, χ^2^ = 0.066). Although the EasyNAT detected 97.78% (44/45) of dPCR-positive cases, the statistical difference was not significant (*P* = 0.898, χ^2^ = 0.017).

**TABLE 3:** The detection outcomes of different methods for 137 clinical samples

Sample number	Outcomes of testing			
	EasyNAT Malaria	Smear	RDT	dPCR
90	−	−	−	−
42	+	+	+	+
2	+	−	+	+
2	+	−	−	+
1	−	−	−	+

For malaria detection, the total concordance rate of microscopic examination and the EasyNAT Malaria was 97.08%. Compared with the gold standard (microscopy), the positive and negative concordance rates of the EasyNAT were 97.1% and 95.79%, respectively. Compared with commercial malaria rapid diagnostic kits, the positive, negative, and total concordance rates of the EasyNAT Malaria Assay were 100%, 97.85%, and 98.54%, respectively (Table 4).

**TABLE 4:** Detection results of clinical samples of the EasyNAT Malaria Assay

		EasyNAT Malaria				
		Positive	Negative	Positive concordance rate	Negative concordance rate	Total concordance rate
Microscopic examination	Positive	42	0	100%	95.79%	97.08%
	Negative	4	91			
RDT	Positive	44	0	100%	97.85%	98.54%
	Negative	2	91			
dPCR	Positive	45	1	97.83%	100%	99.27%
	Negative	0	91			

Among 55 clinically suspicious samples, two samples turned out to be positive under EasyNAT tests (Tt value: 15.33 and 13.83; 13.5 and 13.5, separately) and dPCR. By contacting the patients and taking their medical history, it was found that these two patients had self-medicated with antimalarial drugs overseas before seeking medical attention in our hospital. As a result, there may be extremely low concentrations of malaria infection or malaria nucleic acid residues in their body. Therefore, both microscopic examination and RDT tests failed to find positive traces. It has been reported that the limit of detection of the traditional thin blood smear is no more than 4–20 parasites/μL, and qualitative antigen tests showed better sensitivity at a parasitemia of 101 –1,000 parasites/μL (<0.02%) (6, 7). The results indicated that the EasyNAT Malaria Assay might have an enhanced diagnostic accuracy in malaria patients who have taken anti-malarial medication before their clinical appointment.

As for dPCR, the total, positive, and negative concordance rates of the EasyNAT Malaria Assay were 99.27%, 97.83%, and 100%, respectively (Table 4). For the samples positive only under dPCR, the quantitative results by dPCR were indeed below the LOD of the EasyNAT validated in this study. According to the data from this study and our previous research (5) and considering the differences in sample type (whole blood for the EasyNAT and extracted DNA for dPCR) and volume added during testing, the standardized parasite count required for each positive test is at least two parasites/test for EasyNAT and 0.8 parasites/test for dPCR approximately.

## DISCUSSION

The EasyNAT Malaria Assay exhibited good performance in clinical samples with excellent sensitivity and specificity, and the whole process is time-saving, accurate, and easy to operate. The amplification and nucleic acid products were fully enclosed in a sealed cartridge tube, avoiding biosecurity contamination. By utilizing the existing Ustar nucleic acid analyzer, whether it is two, four, eight, or 16 channels, all the clinical samples can be tested on the spot in an emergency laboratory.

Thus far, most commercial rapid diagnostic tests have targeted malaria-specific protein antigens or enzymes (8), and no commercial products are yet available for detecting *Plasmodium* species based on nucleic acid testing. Recently, in 2020 and 2021, two teams from India and Peru reported a portable magneto-optical detection device for *Plasmodium* diagnosis called Gazelle (9, 10). It has good performance in diagnosing low parasitemia with an infection concentration >200 parasites/μL, and pfhrp2/3 deletion mutant parasites can also be detected effectively (9). Compared with Gazelle, our new rapid molecular detection method has a better LOD and clinical performance. It provides a promising technique for laboratory staff to screen suspected patients. After a simple instrumental interpretation, limited time and effort can be better used to read highly suspected blood smears because it is still regarded as the standard method.

However, high-sensitivity detection methods can also pose some problems. For those patients who have self-medicated with antimalarial drugs and exhibited negative results through microscopic examination and RDT tests but positive results under the EasyNAT Malaria Assay, it is difficult to determine whether the patient has parasitemia and whether further medication is needed, considering that those positive amplifications can possibly be caused by treatment inducing nucleic acid fragments. Additionally, compared with conventional laboratory assessments, especially light microscopy, the cost of the EasyNAT Malaria is definitely higher, and it requires substantial capital upfront for the purchase of instruments, making it difficult to be affordable and suitable for low-resource settings.

The biggest shortcoming of the current study is that it can only report malaria-positive and malaria-negative results without distinguishing between different species. Given that various malaria species require different treatment regimens, the EasyNAT Malaria Assay is more suitable for the initial management of ER patients. Further classification is still required for confirmed patients. In addition, because the current study was limited by the number of malaria-positive samples and low prevalence, it will be necessary to expand the collection to a larger sample size for future studies, especially for *P. vivax*, *P. malariae*, and *P. ovale*, and validate in other geographic areas, especially in highly malaria-endemic regions.
